# Bevacizumab for ocular neovascular diseases: a systematic review

**DOI:** 10.1590/S1516-31802009000200006

**Published:** 2009-07-07

**Authors:** Regis Bruni Andriolo, Maria Eduarda Puga, Rubens Belfort, Álvaro Nagib Atallah

**Affiliations:** 1 MSc. Affiliated researcher at Brazilian Cochrane Center and postgraduate student at the Discipline of Emergency Medicine and Evidence-Based Medicine, Department of Medicine, Universidade Federal de São Paulo - Escola Paulista de Medicina (Unifesp-EPM), São Paulo, Brazil.; 2 MD, PhD. Full professor of the Discipline of Ophthalmology, Department of Ophthalmology, Universidade Federal de São Paulo - Escola Paulista de Medicina (Unifesp-EPM), São Paulo, Brazil.; 3 MD, PhD. Full professor of the Discipline of Emergency Medicine and Evidence-Based Medicine, Department of Medicine, Universidade Federal de São Paulo - Escola Paulista de Medicina (Unifesp-EPM), São Paulo, Brazil.

**Keywords:** Angiogenesis inhibitors, Retinal neovascularization, Corneal angiogenesis, Macular degeneration, Review., Inibidores da angiogênese, Neovascularização retiniana, Neovascularização da córnea, Degeneração macular, Revisão.

## Abstract

**CONTEXT AND OBJECTIVE:**

Many eye diseases involve increased local levels of vascular endothelial growth factor (VEGF), and there are several therapeutic strategies for them. Thus, the aim of this study was to evaluate the effectiveness and safety of bevacizumab for treating eye diseases involving increased local levels of VEGF, as the assumed pathophysiological mechanism.

**DATA SOURCES:**

The following databases were systematically searched for evidence: PubMed, CENTRAL (Cochrane Library), Literatura Latino-Americana e do Caribe em Ciências da Saúde (Lilacs) and reference lists, without language restrictions. Only randomized controlled trials were included. The primary outcome of interest was visual acuity, irrespective of the evaluation method.

**DATA SYNTHESIS:**

A total of 667 eyes in nine randomized trials were included. Meta-analysis showed that the proportion of patients with age-related macular degeneration who presented improvements from baseline regarding best-corrected visual acuity was higher among those treated with bevacizumab than among those in the photodynamic therapy group (risk ratio, RR, 0.49; 95% confidence interval, CI, 0.31 to 0.78; P = 0.01).

**CONCLUSIONS:**

The evidence available demonstrates that bevacizumab alone or combined with other treatments is more effective than other options, including photodynamic therapy, focal photocoagulation and triamcinolone. The use of bevacizumab instead of photodynamic therapy could reduce treatment costs by more than 99% and could significantly increase access to treatment. However, long-term studies are still needed in order to reduce uncertainty concerning the safety of this medication for all ocular neovascular diseases in which bevacizumab has the potential to improve visual acuity.

## RESUMO

**CONTEXTO E OBJETIVOS:**

Muitas doenças oculares envolvem o aumento dos níveis locais de fator de crescimento do endotélio vascular (FCEV), uma diversidade de estratégias terapêuticas para tais condições. Assim, o objetivo do presente estudo é avaliar a efetividade e a segurança de bevacizumabe para o tratamento de pacientes com doença ocular que envolva o aumento dos níveis locais de FCEV, como mecanismo patofisiológico assumido.

**FONTE DAS INFORMAÇÕES:**

Foi realizada busca sistemática pelas evidências disponíveis nas seguintes bases de dados da eletrônicas: PubMed, CENTRAL (The Cochrane Library), Literatura Latino-Americana e do Caribe em Ciências da Saúde (Lilacs), além de referências bibliográficas de estudos relevantes, sem restrições de língua. Foram incluídos apenas ensaios controlados e aleatórios. Acuidade visual, independentemente do método de avaliação, foi considerada o desfecho primário de interesse.

**SÍNTESE DOS DADOS:**

Foi incluído um total de 667 olhos testados em nove ensaios clínicos aleatórios. A metanálise demonstrou que a proporção de pacientes com degeneração macular relacionada à idade que melhoraram a acuidade visual foi maior entre os tratados com bevacizumabe do que entre os pacientes em terapia fotodinâmica (risco relativo [RR] 0.49, 95% intervalo de confiança [IC] 0,31 a 0,78, P = 0,01).

**CONCLUSÕES:**

A evidência disponível demonstra que bevacizumabe isolado ou combinado com outras terapias é mais eficaz que terapia fotodinâmica, fotocoagulação focal e triancinolona. O uso de bevacizumabe em vez da terapia fotodinâmica poderia reduzir os custos do tratamento em mais de 99% e aumentar significativamente o acesso ao tratamento. Entretanto, o aspecto de segurança do fármaco ainda necessita ser avaliado por estudos em longo prazo com todas as doenças neovasculares em que bevacizumabe tenha o potencial de melhorar acuidade visual.

## INTRODUCTION

Many eye diseases and problems associated with ocular structures and tissues involve a single pathophysiological mechanism relating to increased local levels of vascular endothelial growth factor (VEGF) and consequently to neovascularization.^1^ Such diseases can include age-related macular degeneration, affecting 5% to 27% of health-plan beneficiaries, proliferative diabetic retinopathy, affecting 14.5% to 25.6% of patients with diabetes mellitus,^2^ and other less prevalent diseases, such as Stevens-Johnson syndrome, with 2.2 to 7.1 cases per million inhabitants.^3^

**Figure 1.**
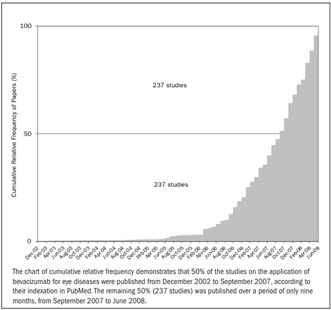
Papers reporting on bevacizumab for ocular diseases published per month in the PubMed database.

Bevacizumab, a monoclonal antibody that binds to all VEGF isoforms, was developed to treat colorectal cancer,^4^ and its use for ocular diseases has not yet been approved by the United States Food and Drug Administration (FDA).^5^ Nevertheless, use of bevacizumab has been supported by Medicare in the United States since July 2006, thus suggesting that the information available is sufficient to allow bevacizumab to be purchased and included in the management of neovascular macular degeneration.^6^ A growing number of researchers are making information available regarding the use of bevacizumab for ocular diseases. Figure 1 demonstrates the number of papers on this subject that have been published since the first one in 2002.

This scenario clearly indicates the need to conduct a systematic review of randomized clinical trials, in order to reduce the uncertainties and establish guidelines for future randomized clinical trials that test hypotheses about bevacizumab for patients diagnosed with ocular neovascular diseases.

## METHODS

### Data sources and searches

The following databases were searched: Medical Literature Analysis and Retrieval System Online (Medline) (1966 to June 2008), Controlled Trials Register of the Cochrane Collaboration (2008, issue 2) and Literatura Latino-Americana e do Caribe em Ciências da Saúde (Lilacs) (1982 to June 2008). A general search strategy was used, with adaptations to the characteristics of each database, to identify studies on bevacizumab for ocular diseases that involved increased local levels of VEGF. Descriptors and synonyms for the intervention of interest (Avastin^®^, bevacizumab) and for the clinical conditions for which this medication potentially could be indicated were used (e.g. age-related macular disease, corneal neovascularization, retinal angiomatous proliferation or angiogenic retinal diseases, among others).

Study selection

We planned to include only randomized or quasi-randomized clinical trials that tested bevacizumab alone or in association with other strategies available. The clinical condition of interest among the individuals included (of both genders, independent of ethnicity and age) was a diagnosis of ocular diseases or ocular conditions with the same underlying pathophysiological mechanism of increased local levels of VEGF, according to the criteria established by the authors of the primary studies, such as age-related macular disease, corneal neovascularization, retinal angiomatous proliferation or angiogenic retinal diseases, among others. Studies in which the analysis unit was based on the eyes were not included, since there is evidence demonstrating an adverse event (vitritis) associated with bevacizumab in the contralateral eye.^7^

### Data extraction and quality assessment

The methodological quality of the studies included was analyzed independently by two authors (RBA and ANA) according to the risk of bias (low, moderate or high risk) relating to the following items: selection bias, performance bias, detection bias and attrition bias.^8^

The outcome of interest for this review was visual acuity, as measured by any validated evaluation instrument (e.g. Snellen acuity charts, Vernier acuity, Early Treatment Diabetic Retinopathy Study chart and others). Adverse events, e.g. ocular or systematic hypertension, ocular toxicity, local inflammation, retinal detachment, vitreous hemorrhage, corneal abrasions, lens injury and thromboembolic events, were assessed. Surrogate outcomes, such as central foveal thickness, fluorescein angiography and others, were not considered in this analysis. When the outcome was reported in more than one paper, these results were combined in meta-analyses using Review Manager 5.0,^9^ which was developed by the Cochrane Collaboration. Dichotomous data were calculated as risk ratios (RR) with 95% confidence intervals (95% CI). The estimated effects were combined using the random effect model,^10^ which considers outliers to be related to heterogeneities of a clinical and methodological nature, even when they are unknown. Continuous data were expressed as means and standard deviations, and weighted mean differences (WMD) were calculated in relation needed to the 95% CI level. For continuous data, the numerical information needed to perform such analysis was composed of the total number of patients and the mean and standard deviation, per comparison group. For dichotomous data, the numerical information needed to perform such analysis was composed of the total number of patients and the number of events, per comparison group. The reviewers grouped the data as a function of co-interventions. Statistical heterogeneity between the study results was evaluated using the inconsistency test (I^2^), such that inconsistency was considered present when I^2^ > 50%.^11^^,^^12^

## RESULTS

### Description of studies with potential for inclusion

**Figure 2.**
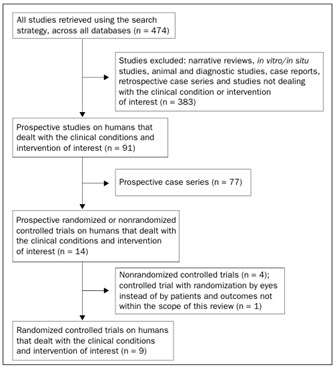
Flow chart of studies, from databases to inclusion in the systematic review.

Out of the 474 studies retrieved through the search strategy, only nine satisfied the preestablished inclusion criteria (Figure 2). A Cochrane systematic review covering all anti-VEGF therapies for age-related macular degeneration was found, but the authors had not included studies testing bevacizumab by the time the present systematic review was finished.^13^ One randomized study was excluded because its outcomes did not align with the purpose of this review and because it allocated the two eyes of each patient to different comparison groups.^14^ The nine studies included in the present review yielded a total of 667 randomized eyes, as shown in Table 1.^15^^,^^16^^,^^17^^,^^18^^,^^19^^,^^20^^,^^21^^,^^22^^,^^23^

Three studies included patients with diagnoses of diabetic macular edema.^17^^,^^20^^,^^21^ One study included patients with diagnoses of clinically significant macular edema who had not responded to earlier subsequent photocoagulation therapy.^15^ Three studies included patients with diagnoses of age-related macular degeneration.^18^^,^^19^^,^^23^ One study tested bevacizumab on patients with subfoveal choroidal neovascularization associated with age-related macular degeneration.^16^ Finally, there was one study that included patients with diagnoses of proliferative diabetic retinopathy.^22^

With regard to the origin of the papers, two studies were conducted in Brazil,^11^^,^^22^ two in Iran,^15^^,^^21^ one in Lebanon,^16^ one in the United States,^17^ one in Austria,^23^ one in Croatia^19^ and one in Germany.^18^ None of these studies mentioned any financial support from pharmaceutical companies.

**Table 1.** Comparison groups and bevacizumab regimen tested in each study

Reference	Intervention		Description available in the report
15	a.	bevacizumab	3 ´1.25 mg at 6-week intervals
	b.	bevacizumab triamcinolone	3 ´ 1.25 mg at 6-week intervals 2 mg (at first session)
	c.	sham	syringe without needle pressed against the conjunctiva and sclera
16	a.	bevacizumab	2.5 mg ´ mean of 2.4 treatments
	b.	photodynamic therapy	mean of 2.3 treatments with verteporfin
17	a.	focal photocoagulation	single dose
	b.	bevacizumab	1.25 mg on entry and after 6 weeks
	c	bevacizumab sham injections	1.25 mg on entry one per week
	d.	bevacizumab focal photocoagulation	1.25 mg on entry and after 6 weeks at week 3
	e.	bevacizumab	2.5 mg upon entry and after 6 weeks
18	a.	bevacizumab	1.0 mg (single dose)
	b.	triamcinolone photodynamic therapy	4.0 mg (single dose) verteporfin (single dose)
	c.	triamcinolone photodynamic therapy	4.0 mg verteporfin
19	a.	bevacizumab	1.25 mg (single dose)
	b.	photodynamic therapy	verteporfin (single dose)
	c.	bevacizumab photodynamic therapy	1.25 mg within 1 hour after verteporfin verteporfin (single dose)
20	a.	triamcinolone	4.0 mg (upon entry)
	b.	bevacizumab	1.5 mg (upon entry)
21	a.	bevacizumab	1.25 mg (upon entry)
	b.	bevacizumab triamcinolone	1.25 mg (upon entry) 2.0 mg (upon entry)
	c.	macular laser photocoagulation	single dose
22	a.	bevacizumab panretinal photocoagulation	1.5 mg, week 3 weeks 1 and 3
	b.	panretinal photocoagulation	weeks 1 and 3
23	a.	bevacizumab	1.0 mg (mean of 4.5 treatments)
	b.	photodynamic therapy triamcinolone	verteporfin (mean of 1.9 treatments) 4.0 mg (mean of 1.9 treatments)

### Methodological quality of the studies included

### Selection bias

Four studies were considered to present low risk of selection bias, since they were properly conducted with regard to this factor.^15^^,^^19^^,^^21^^,^^22^ All other studies were considered to present moderate risk because, although their allocations were random, the methods used for generating randomization were not stated. One study allocated the eyes with the worst visual acuity to receive panretinal photocoagulation in association with bevacizumab (eight eyes), while all other eyes were treated with panretinal photocoagulation alone (eight eyes), among patients who were at high risk of proliferative diabetic retinopathy in both eyes. For this reason, this study was considered to present a high risk of systematic error.^22^ Separate data on the group that was randomly allocated were not available.

### Performance bias

With the exception of three studies^15^^,^^17^^,^^21^ that made use of control groups receiving sham injections or laser, none of the other studies reported any care taken to prevent performance bias.

### Detection bias

The majority of the studies reported that the evaluators were unaware of the groups to which each patient (eyes) was allocated.^15^^,^^16^^,^^19^^,^^21^^,^^22^ Thus, only these five studies were considered to present a low risk of detection bias. All the others were considered to present a high risk of systematic error.

### Attrition bias

None of the studies reported any substantial losses from their samples, either overall or among their comparison groups. For this reason, all the studies were considered to present low risk of attrition bias.

### Outcome measurements

### Best-corrected visual acuity

**Figure 3.**
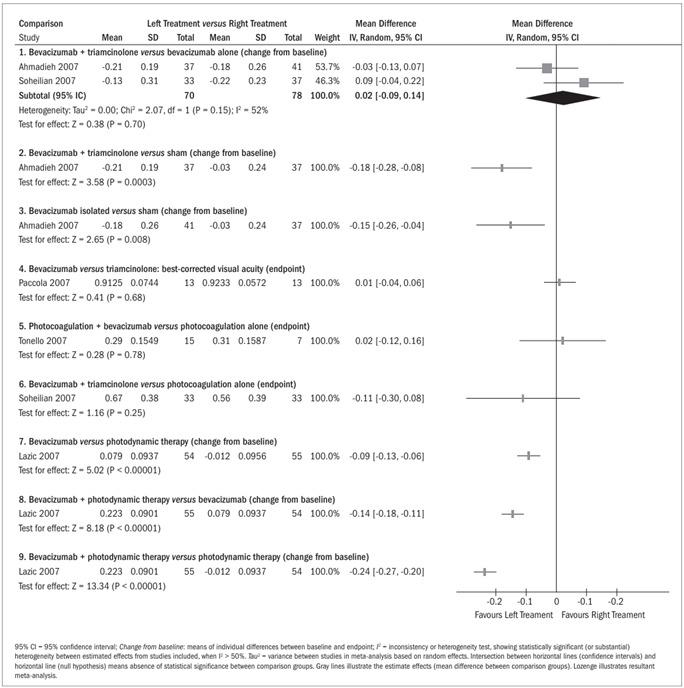
Mean best-corrected visual acuity (logMAR: endpoint and change from baseline).

Bevacizumab alone was shown to be better than the association of bevacizumab and with triamcinolone for best-corrected visual acuity (logMAR, change from baseline), but without a statistically significant mean difference (MD) (MD, 0.02; 95% CI, - 0.09 to 0.14; P = 0.70]. However, in one study,^15^ the estimate effect favored the group treated with bevacizumab in association with triamcinolone (MD, -0.03; 95% CI, -0.13 to 0.07) (Figure 3, comparison 1; two studies^15^^,^^21^). Comparisons between bevacizumab (both in association with triamcinolone and alone) and sham injections demonstrated statistically significant MD in favor of the bevacizumab groups (MD, - 0.18; 95% CI, - 0.28 to -0.08; P = 0.0003 and MD, - 0.15; 95% CI, - 0.26 to - 0.04; P = 0.008, respectively) (Figure 3, comparisons 2 and 3; one study^15^).

A statistically nonsignificant MD was observed for best-corrected visual acuity (logMAR, endpoint), slightly favoring the group treated with bevacizumab over the group treated with triamcinolone (MD, 0.01; 95% CI, - 0.04 to 0.06; P = 0.68) (Figure 3, comparison 4; one study^20^). On the other hand, panretinal photocoagulation alone was better, but without statistical significance, than when combined with 1.5 mg bevacizumab (MD, 0.02; 95% CI, - 0.12 to 0.16; P = 0.78) (Figure 3, comparison 5; one study^22^). Bevacizumab (1.25 mg) in association with triamcinolone was also shown to be better than laser photocoagulation alone (MD, - 0.11; 95% CI, - 0.30 to 0.08; P = 0.25) (Figure 3, comparison 6; one study^21^). For these two comparisons, the MD between the groups were not statistically significant. On the other hand, comparison between bevacizumab alone and triamcinolone in association with photodynamic therapy showed a statistically significant difference in favor of bevacizumab with regard to the endpoint of best-corrected visual acuity (P < 0.005) in one study. However, the available estimated effect was not appropriate for inclusion in a forest plot.^18^

Bevacizumab alone was shown to be better than photodynamic therapy for best-corrected visual acuity (logMAR, change from baseline), with a statistically significant MD (MD, - 0.09; 95% CI, - 0.13 to - 0.06; P < 0.00001) (Figure 3, comparison 7; one study^19^). Bevacizumab in association with photodynamic therapy was shown to be better than both bevacizumab alone and photodynamic therapy alone, with statistically significant MD (MD, -0.14; 95% CI, - 0.18 to - 0.11; P < 0.00001 and MD, - 0.24; 95% CI, - 0.27 to - 0.20; P < 0.00001, respectively) (Figure 3, comparisons 8 and 9; one study^19^).

### Patients whose best-corrected visual acuity decreased (logMAR, irrespective of authors’ criteria)

**Figure 4.**
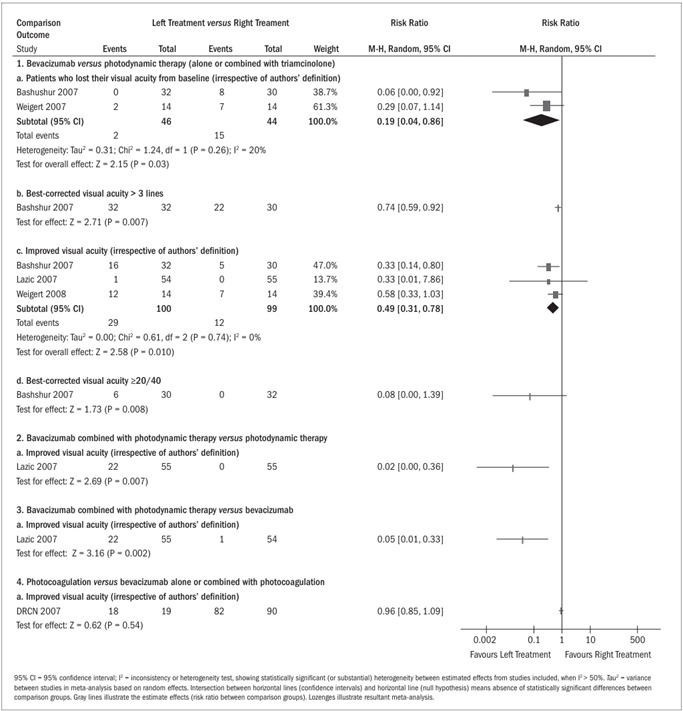
Risk ratio for dichotomous outcomes.

There was a statistically significant greater proportion of patients whose visual acuity was not reduced by more than three lines in the group treated with bevacizumab (2/46), compared with the group treated with photodynamic therapy (15/44), with a risk ratio (RR) of 0.19 (95% CI, 0.04 to 0.86; P = 0.03). It was necessary to change three patients (NNT, number needed to treat) from photodynamic therapy to bevacizumab to avoid an additional patient presenting any losses in visual acuity (95% CI, 2 to 7) (Figure 4, comparison 1a; two studies^16^^,^^23^).

### Patients who achieved visual acuity > three lines

The proportion of patients who presented visual acuity greater than three lines was higher and statistically significant in the group treated with bevacizumab (32/32) than in the group treated with photodynamic therapy (22/30) (RR, 0.74; 95% CI, 0.59 to 0.92; P = 0.007 and NNT = 4; 95% CI, 2 to 10) (Figure 4, comparison 1b; one study^16^).

### Improvements from baseline in best-corrected visual acuity

A greater proportion of patients presented increased visual acuity (irrespective of authors’ criteria) in the group treated with bevacizumab (29/100) than in the group treated with photodynamic therapy alone or combined with triamcinolone (12/99) (RR, 0.49; 95% CI, 0.31 to 0.78; P = 0.003 and NNT = 4; 95% CI, 1 to 4) (Figure 4, comparison 1c; three studies^16^^,^^19^^,^^23^). However, bevacizumab combined with photodynamic therapy was shown to benefit more patients (22/55) than did photodynamic therapy alone (0/55), with RR of 0.02 (95% CI, 0.00 to 0.36; P = 0.007 and NNT = 2; 95% CI, 2 to 4) or bevacizumab alone (1/54), with RR of 0.05 (95% CI, 0.01 to 0.33; P = 0.002 and NNT = 3; 95% CI, 2 to 4) (Figure 4, comparisons 2 and 3, respectively; one study^19^). There was no statistically significant difference between focal photocoagulation alone (18/19) and bevacizumab alone or combined with focal photocoagulation (82/90), with RR of 0.96 (95% CI, 0.85 to 1.09; P = 0.54) (Figure 4, comparison 4; one study^17^).

### Best-corrected visual acuity ≥ 20/40

A greater proportion of patients presented visual acuity ≥ 20/40 in the group treated with bevacizumab (6/30) than in the group treated with photodynamic therapy (0/32), without obtaining statistical significance in estimating this effect (RR, 0.08; 95% CI, 0.00 to 1.39; P = 0.08 and NNT = 5; 95% CI, 3 to 25) (Figure 4, comparison 1d; one study^16^).

### Adverse events

Although the adverse events potentially associated with bevacizumab were of diverse types,^24^ the most common adverse events associated with bevacizumab, irrespective of whether alone or not, were: moderate anterior chamber reaction (19%),^15^ transient anterior chamber reaction (16%),^21^ iris neovascularization (11%),^15^ subconjunctival hemorrhage,^22^ posterior vitreous detachment (15%)^19^ and foreign body sensation.^22^ More details about other adverse events are shown in Table 2.

**Table 2.** Adverse events

Adverse events	Comparison groups								
	Bv % (n/N)	Pg + Bv % (n/N)	Tr + Bv % (n/N)	PDT + Bv % (n/N)	Sham % (n/N)	Pg % (n/N)	PDT % (n/N)	Tr % (n/N)	PDT + Tr % (n/N)
Anemia^17^	4 (4/90)	-	-	-	-	5 (1/19)	-	-	-
Cataract progression^19^	7 (4/54)	-	-	6 (3/50)	-	-	0 (0/52)	-	-
Congestive heart failure^17^	1 (1/90)	-	-	-	-	0 (0/19)	-	-	-
Death^17^	2 (2/90)*	-	-	-	-	0 (0/19)	-	-	-
Elevation of blood pressure^17^	1 (1/90)	-	-	-	-	0 (1/19)	-	-	-
Endophthalmitis^17^	1 (1/90)	-	-	-	-	0 (0/19)	-	-	-
Foreign Body sensation^22^	-	13 (2/15)	-	-	-	0 (0/7)	-	-	-
Intraocular pressure rise^15^^,^^17^^,^^20^^,^^21^^,^^23^	4 (1/124)	-	8 (6/70)	-	0 (0/37)	0 (0/52)	-	7 (1/13)	14 (2/14)
Iris neovascularization^15^	22 (9/41)	-	0 (0/37)	-	0 (0/37)	-	-	-	-
Marked anterior chamber reaction^15^	2 (1/41)	-	3 (1/37)	-	0 (0/37)	-	-	-	-
Moderate anterior chamber reaction^15^	19 (8/41)	-	19 (7/37)	-	0 (0/37)	-	-	-	-
Myocardial infarction^17^	2 (2/90)	-	-	-	-	0 (0/19)	-	-	-
Peripheral vascular disease^17^	1 (1/90)	-	-	-	-	10 (2/19)	-	-	-
Pigment epithelial tears^19^	3 (3/54)	-	-	0 (0/50)	-	-	0 (0/52)	-	-
Posterior vitreous detachment^19^	15 (8/54)	-	-	8 (4/50)	-	-	0 (0/52)	-	-
Progression of fibrous proliferation^15^	2 (1/41)	-	0 (0/37)	-	0 (0/37)	-	-	-	-
Subconjunctival hemorrhage^22^	-	47 (7/15)	-	-	-	0 (0/7)	-	-	-
Syncope^17^	1 (1/90)	-	-	-	-	0 (0/19)	-	-	-
Transient anterior chamber reaction^21^	19 (7/37)	-	12 (4/33)	-	-	0 (0/33)	-	-	-
Transient intraocular pressure rise^17^	1 (1/90)	-	-	-	-	0 (0/19)	-	-	-
Vitreous hemorrhage^15^	0 (0/41)	-	3 (1/37)	-	0 (0/37)	-	-	-	-
Worsened renal function^17^	3 (3/90)	-	-	-	-	0 (0/19)	-	-	-

The percentages were obtained from all studies reporting at least one event in the bevacizumab group (irrespective of whether combined with other options or alone). Bv = Bevacizumab; Pg = Photocoagulation; Tr = Triamcinolone; PDT = Photodynamic therapy. *Reported causes of death were myocardial infarction and pancreatic cancer.

## DISCUSSION

Based on the primary outcome that we planned to analyze in this systematic review, the results showed that intraocular use of bevacizumab alone was better than photodynamic therapy (alone or combined with triamcinolone) for patients with subfoveal choroidal neovascularization associated with age-related macular degeneration, choroidal neovascularization due to age-related macular degeneration and age-related macular degeneration alone, as reported by the authors of the primary studies. Bevacizumab combined with photodynamic therapy was shown to be better than photodynamic therapy alone and bevacizumab alone for patients diagnosed with choroidal neovascularization due to age-related macular degeneration. On the other hand, photocoagulation was shown to be better than bevacizumab (alone or combined with photocoagulation) for patients diagnosed with diabetic macular edema, but this finding was not statistically significant, probably because of the small sample size.

Despite the sensitivity of the search strategy used and the large number of published papers on this subject (474 studies), only nine trials satisfied the strict inclusion criteria. These studies presented the minimum methodological rigor appropriate for this type of question (i.e. regarding disease treatment) since they were clinical trials with random allocation.^25^ However, it needs to be noted that the allocation of participants in the subgroup at high risk of proliferative diabetic retinopathy in both eyes was not random in one study.^22^ Furthermore, in one study,^23^ the nature of the interventions allowed concealment of the allocation and blinding of patients and therapists regarding the use of bevacizumab or triamcinolone, but blinding would not be operationally easy for photodynamic therapy. It was decided to include these studies, given the lack of high-quality controlled studies on the application of bevacizumab for treating ocular diseases at the time when this systematic review was implemented.

The fact that Mirshahi et al.^14^ was not included in this review deserves attention. Today, the internal validity of studies in which the eyes of a single patient are allocated to different groups is considered to be unclear. This is illustrated by the existence of contradictory studies. Indeed, there is evidence from studying the pharmacokinetic aspects of bevacizumab that the possibility of contralateral effects (through systemic absorption of the drug by the contralateral eye) is remote.^26^ On the other hand, there is evidence demonstrating the opposite, through manifestations of adverse events associated with bevacizumab in the contralateral eye.^7^ Therefore, it seems sensible for the time being for researchers to choose types of allocation other than involving different treatments for each eye of the same individual.

The present scenario is that, taken together, the studies that have been published are still of an exploratory nature, given the diversity of comparisons, co-interventions, dosages and variables of interest (outcome measurements), along with the variety of ways of reporting these variables. Thus, it is recommended that the specialists within this field should come to a consensus regarding which outcomes are relevant and how these should be analyzed.

It is possible to estimate the costs associated with bevacizumab. Bashur et al. found that an average of 2.4 treatments with 2.5 mg bevacizumab per patient was needed to treat persistent subretinal fluid or cystic macular disease, and that 2.3 treatments with photodynamic therapy were needed when leakage from choroidal neovascularization was present on fluorescein angiography over a six-month period.^16^ The gross cost of bevacizumab is about USD 5.5 per mg (United States dollars).^27^ The assumed cost of photodynamic therapy with verteporfrin, or Visudyne^®^ is USD 3,000.00.^28^ The meta-analysis demonstrated that, in order to improve visual acuity in one patient, 3.45 patients (29/100) would have to be treated with Bevacizumab and 8.25 patients (12/99) with photodynamic therapy (Figure 4, comparison 1c). The estimated expense would be USD 113.85 for bevacizumab and USD 56,925.00 for photodynamic therapy. Considering the findings of Brown et al.,^29^ an average of 11 treatments of 0.5 mg ranibizumab per patient would have to be administered, at a cost of USD 3,900.00 per mg.^27^ To improve visual acuity, defined as ≥ 15 letters at 12 months, 2.48 patients (56/139) would have to be treated with ranibizumab. The estimated cost would be USD 53,196.00.

Results from future trials may provide more information about the wide variety of types of outcome measurements (including adverse events), comparisons and co-interventions, such as photodynamic therapy, laser photocoagulation, triamcinolone and vitrectomy.
